# A landscape analysis and one health approach to an invasive species pathway: Pet and aquaria trade in the eastern Caribbean

**DOI:** 10.1016/j.onehlt.2024.100942

**Published:** 2024-11-21

**Authors:** Kirk O. Douglas, Diana Francis, Naitram Ramnanan

**Affiliations:** a Centre for Biosecurity Studies, The University of the West Indies, Cave Hill BB11000, Barbados; b Inter-American Institute for Cooperation on Agriculture (IICA), 59 Gordon Street St., Augustine Tunapuna 331323, Trinidad and Tobago; c CAB International (CABI), 59 Gordon Street St., Augustine Tunapuna 331323, Trinidad and Tobago

**Keywords:** Invasive alien species, Pet trade, One health, Biosecurity, Caribbean, Risk prioritization

## Abstract

**Background:**

The pet and aquaria trade is a pathway for the introduction of invasive alien species (IAS) into sensitive Caribbean ecosystems. This study aims to assess the impact of this trade on IAS management in the Caribbean.

**Methods:**

A multipronged approach was used, involving stakeholder engagement, trade flow analysis, questionnaires, a regional IAS workshop, and a One Health Invasive Alien Species Prioritization (OHIASP) method, to examine the pet and aquaria trade in Barbados and the Organisation of Eastern Caribbean States (OECS). These methods allow for a comprehensive tool necessary to prioritise IAS challenges worldwide particularly in Barbados and the OECS.

**Results:**

The study assessed seven Caribbean countries: Barbados, St. Lucia, Grenada, Dominica, St. Kitts and Nevis, St. Vincent and the Grenadines, and Antigua and Barbuda. Barbados reported the highest annual import values for pets (USD $371,604) and aquaria (USD $450,860) using data from 2016 to 2020. The species range was very narrow and likely a reflection of data collection systems. Trinidad and Tobago was the primary regional source for pet and aquaria imports. In total, 35 IAS were chosen for prioritization. Based on their weighted score, prioritized IAS were ranked in order of relative importance using a one-to-five selection scale. A priority list of 13 IAS was identified from the pet/aquaria imported into Barbados and the OECS.

**Conclusions:**

This marks the first ever study using an OHIASP tool for examining, quantifying and ranking IAS risks in pet and aquaria trade pathways. This can assist zoonotic disease risk prioritization where necessary. Effective IAS management in the Caribbean requires multipronged approaches, data and information systems that integrate indigenous knowledge, leverage digital tools, and build community ownership, to overcome inherent regional vulnerabilities.

## 1 Introduction

Invasive alien species (IAS) are plants, animals, pathogens, and other life forms introduced by humans that spread outside their natural habitats [1]. Impacts on biodiversity include decline or extinction of indigenous species through competition, predation, hybridisation and transmission of pathogens, and the disruption of local ecosystems and ecosystem functions [2]. IAS invasions continue as a consequence of expanding global trade and increased international movement of humans, biological material and other commodities [[2], [3], [4]].

The pet and aquaria trade continues to receive growing interest globally, and is a well documented pathway of IAS into countries within varied regions across the globe [5,6]. Article 8 h of the United Nations Convention on Biological Diversity (CBD) explicitly references IAS as problematic, and calls on signatory nations to “*prevent the introduction of, control or eradicate those alien species which threaten ecosystems, habitats or species*” [7]. The literature confirms that a weakened ecosystem impacted by IAS makes it more vulnerable to invasions [8,9]. Numerous IAS introductions can result in cumulative effects as more introductions, whether of the same or new species, lead to a greater likelihood of establishment and spread [10]. Prioritization of biosecurity for IAS must be managed within and across stages of the invasion process, i.e., before (pre-border), at the border, and after (post-border) invasion [11,12].

Island territories, occupy only 5.5 % of the land surface, and are host to almost 15 % of terrestrial species [13]. For small island developing states (SIDS), IAS introductions and their subsequent establishment have had varied ecological and socioeconomic impacts, with over USD 21 billion spent on global biodiversity and conservation [14,15]. In the Caribbean several examples of negative impacts of animal IAS on agriculture have occurred [14,16]. The Caribbean remains vulnerable to various natural hazards including hurricanes, floods, wildfires, and natural disasters which cause extensive damage to infrastructural damage potentially to terrestrial and aquatic animal enclosures [17]. In post-disaster recovery the need for biosecurity and IAS management is frequently overlooked, resulting in lapses in containment and monitoring [18,19]. Participatory approaches to acquire and mainstream traditional or local knowledge and critically reviewing cases with invasive species linked to the pet and aquaria trade are essential for effective and sustainable IAS management [20]. A One Health strategy is crucial to addressing these economic, agricultural and human health risks since there is a lack of thorough regional data on pet and aquaria trade and the inherent risks present therein. There have been several previous studies utilizing the One Health Zoonotic Disease Prioritization (OHZDP) tool for zoonotic diseases [[21], [22], [23]]. The criteria developed with this adapted OHZDP tool were corroborated by previous studies of IAS risk analysis [22,[24], [25], [26], [27], [28], [29], [30], [31], [32], [33]]. Among the published IAS prioritization studies, none have had a distinct focus on the pet and aquaria trade pathway [12,34,35]. Thus, a custom and rationalised approach to IAS prioritization for the pet and aquaria trade in Barbados and the OECS using a modified OHZDP or One Health Invasive Alien Species Prioritization (OHIASP) will be conducted by substituting zoonoses for invasive alien species.

There is a lack of peer-reviewed research data on IAS risk prioritization of the pet and aquaria trade within the Organisation of Eastern Caribbean States (OECS), a collection of 6 different Caribbean countries, and Barbados. For the IAS, pet and aquaria trade data deficient OECS and Barbados countries, a traditional quantitative-based risk and prioritization is just not possible. A mixed methods approach, using interviews and questionnaires, generated the data on the level of awareness of IAS, their impact on biodiversity, size of trade, trade flows, source countries for pets and aquaria fish by importers in Barbados and the OECS. It provided input for a One Health Invasive Alien Species Prioritization (OHIASP) methodology to rank and assess potential IAS risk within the pet and aquaria trade during a regional workshop for future IAS risk management efforts. Zoonoses were substituted by IAS as the focus of the tool and the relevant criteria were changed accordingly to reflect this.

## 2 Mixed methods approaches and use of a modified OHZDP tool for IAS prioritization

This study in Barbados and the OECS, was funded as part of ‘Preventing the COSTS of IAS in Barbados and the OECS” project, to examine pet and aquaria species, not food, agriculture, or research purposes, but animals for companionship and recreational purposes in the pet and aquaria trade. The pet trade is defined as the sale, marketing, import and export of live terrestrial animals and their eggs or other reproductive materials for the use of entertainment, companionship, or leisure. This includes groups of animals such as birds, mammals, amphibians and reptiles. Aquaria trade is defined as the sale, marketing, import and export of live aquatic (fresh/salt water) animals and their eggs or other reproductive materials for the use of entertainment, companionship, or leisure. These include fish and aquaria species.

This research was undertaken through the following methods:

### 2.1 Dialogue with public sector officials for understanding of IAS management of wildlife as pets

**Fig. 1:**
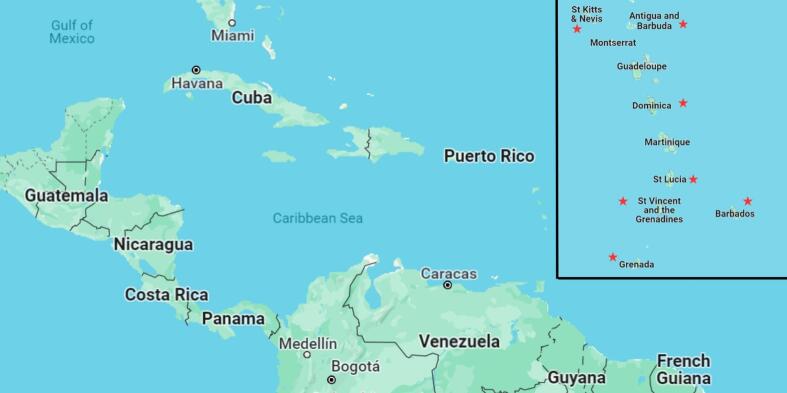
Map of the Eastern Caribbean highlighting countries involved in the study (red star). (For interpretation of the references to colour in this figure legend, the reader is referred to the web version of this article.)

An important initial activity was direct dialogue with key public officials with responsibility for plant and animal quarantine in the Ministries of Agriculture in the six OECS countries and Barbados. The dialogue was to clarify the public sector arrangements for IAS management (identification of specific government agency, their level of interaction with industry stakeholders, regulatory status and capacity gaps for IAS management and the species of greatest interest for priority actions. The dialogue, organised and moderated by Inter-American Institute for Cooperation on Agriculture (IICA), provided a baseline understanding of the current situation with Barbados and the six [6] Eastern Caribbean countries with respect to the status of and capacity for IAS management actions (Fig. 1).

**Fig. 2:**
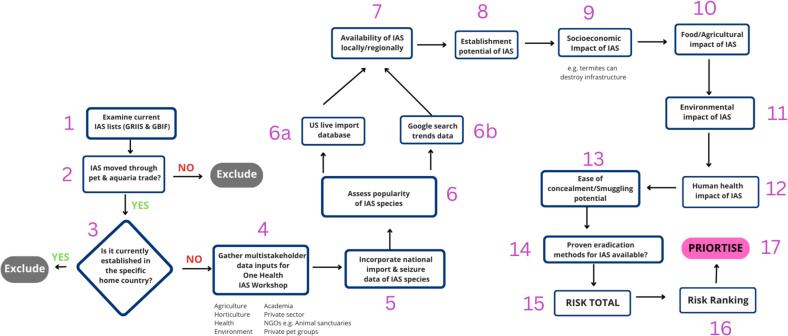
Inclusion and exclusion criteria applied to the invasive alien species (IAS) hazards and IAS risk assessment for the pet and aquaria trade for a specific country. This IAS hazard and risk assessment process started with a consultation of current IAS lists from relevant countries (within the Caribbean and the Americas given the wildlife trading trends) in the Global Register of Introduced and Invasive Species (GRIIS) and or Global Biodiversity Information Facility (GBIF) [1]. The next criteria were knowledge/presence of specific IAS in pet and aquaria trade [2] and its presence or establishment in the specific host country [3]. This was followed by multistakeholder data inputs for One Biosecurity/One Health IAS workshop inclusive of agriculture, horticulture, health, academia, private sector, non-governmental organizations (NGOs e.g. pet sanctuaries) and private pet groups [4], then the incorporation of national import inquiry, inspection and seizure database where available [5]. Next, the popularity of the IAS as a pet was assessed using a combination of US live import database (6 A) and Google search trends data (6B). The next series of criteria were guided by peer reviewed literature and Google Scholar searches including: local/global availability of IAS for pet purchase [7], the establishment potential of the species [8], negative socioeconomic impact, other than agriculture or public health [9], known negative agricultural impacts [10], known negative environmental impacts of IAS [11], negative public health impact (e.g. carriage of zoonotic pathogens) [12], ease of concealment/smuggling potential [13], availability of a proven eradication method [14]. The prioritization process followed with input from local and regional experts. The IAS was scored for each criterion with a minimum of 1 and a maximum of 5, and consensus on each score was reached by workshop participants. The regional IAS workshop was held in St. Kitts & Nevis, 20–22 March 2024 and each participant was invited to actively contribute to each rating and based on consensus among participants the score was finalised and recorded. Note: The eradication method availability rating is as follows: 1- excellent eradiation methods available to 5 – no effective eradiation method available. The cumulative risk was tallied and the total risks for each IAS as a pet was then ranked from highest total [1] to lowest total [13].

### 2.2 Pet and aquaria trade flows analysis

IICA conducted an analysis to understand the potential trade flow patterns under consideration for pet and aquaria purposes. The analysis examined data of animal imports for pet use, for 5 years (2016–2020). The data were extracted from the Harmonized System (HS) in international trade databases and Caribbean Community (CARICOM) Secretariat trade statistics, Chapter HS.01, which covers all live animals, except for fish and crustaceans, molluscs and other aquatic invertebrates which are included in Chapter HS.03, (i.e., 03.01, 03.06, 03.07, or 03.08) [36,37].

### 2.3 In-country stakeholder survey

Listing was based on a desktop identification of who is in the chain for pet and aquaria trade within the selected countries. All listed pet shop operators were contacted by email and telephone and responsive operators were recorded via the survey. Limited stakeholder feedback was undertaken through a questionnaire as the response rate of pet traders was low (Appendix A). This study involved collecting country-speciic information for evidence-based analysis, identifying key stakeholders in the pet and aquaria industry chain, and assessing the efficacy of the current national policy, legal and institutional frameworks for IAS management. IICA coordinated data-gathering processes in each country (Fig. 1), administering questionnaires to key stakeholders and assessing the sector's size. The size of the pet and aquaria trade, type of businesses, and number of businesses were recorded during the stakeholder identification process in 2022.

### 2.4 Regional IAS workshop

A review of existing methodologies and tools to prioritise IAS in pet and aquaria trade led to a draft/preliminary list of thirty-five [35] IAS. Subsequently the IAS workshop, “*Regional Workshop on Preventing the COSTS of Invasive Alien Species in Barbados and the OECS: Successes, lessons learnt, and strategic partnerships in conserving biodiversity”* was held in Saint Kitts and Nevis from 20th-22nd March 2024 (Fig. 1). It was organised in collaboration with CAB International (CABI), IICA, United Nations Environmental Programme (UNEP) and the OECS Commission. It involved 20 participants from multiple disciplines representing CABI, University of the West Indies (Centre for Biosecurity Studies), Environmental Awareness Group (EAG), United Kingdom Royal Society for the Protection of Birds (RSPB), and Animal and Plant Health Agency (APHA) (Table 1). The IAS prioritization criteria were defined, selected and ranked during the regional workshop by attending participants. The modified OHZDP or OHIASP tool and its implementation was conducted during this workshop and all steps were was finalised.

**Table 1:** Participating organizations in the regional workshop on preventing the COSTS of invasive alien species in Barbados and the OECS, 2024.

	Organisation	Number of Participants
National	Department of Environment (Antigua and Barbuda)	1
	Ministry of Environment and National Beautifcation, Green and Blue Economy (Barbados)	2
	Ministry of Sustainable Development, Environment and Climate Action and Constituency Empowerment. (St. Kitts and Nevis)	1
	Ministry of Agriculture, Fisheries, Marine Resources and Cooperatives, Small Business and Entrepreneurship, Sports and The Creative Economy and Entertainment and The Arts (St. Kitts and Nevis)	2
	Ministry of Agriculture, Fisheries, Food Security and Rural Development (St. Lucia)	3
Intergovernmental Organisation	Organisation of Eastern Caribbean States (OECS) Commission	1
Academia	Centre for Biosecurity Studies (University of the West Indies, Cave Hill Campus, Barbados)	1
Non-governmental organisation (NGO)	CAB International (CABI)	4
	United Nations Environmental Programme (UNEP)	1
	Environmental Awareness Group (EAG)	1
	United Kingdom Royal Society for the Protection of Birds	1
Foreign government	Animal and Plant Health Agency (APHA), United Kingdom	1
**TOTAL**		**19**

### 2.5 Adaptation of A one health zoonotic disease prioritization (OHZDP) tool for IAS management

The OECS and Barbados required a blended approach to develop a priority species list of IAS based on available tools, methodologies and knowledge from literature sources. It considered pet shop owners' feedback (key species traded as pets), research on pet and aquaria trade in Trinidad and Tobago and global IAS lists e.g. GRIIS, providing a draft IAS list of 35 species (Appendix 2). Each species was assessed, and criteria were answered using a literature review (horizon scanning), expert knowledge and regional/global data. Next, the relevant scores were recorded in the OHIASP tool [22,24].

A typical OHZDP has only 5 criteria but this was modified during the regional workshop to fit the goal of prioritising IAS. A specific set of suitable criteria were devised and implemented by regional IAS workshop participants to enable screening pet and aquaria species into the OECS and Barbados using an modified OHZDP or OHIASP tool, in a similar manner to previous OHZDP tools [22,24]. These criteria included a) if animal species is known in the global pet/aquaria trade and is available via global online market presence [25,27], b) distribution of species in host or neighbouring country [26], c) availability of animal species in local/regional market (this along with criterion ‘a’ constitute the access of the species) [25,27], d) trending/popularity profile of animal species [27], e) establishment potential and magnitude of potential negative impacts on public health [28,29], f) potential negative agricultural impact of animal species [30], g) potential negative economic impact of animal species [31], h) availability of species eradication methods [33] and i) ease of concealment or smuggling potential [32]. Note that the eradication method availability rating is 1- excellent eradication methods available, to 5 – no effective eradication method available.

## 3 Results

### 3.1 Trade flow analysis

The target data from 2016 to 2020 extracted from the Harmonized System (HS) in international trade databases and CARICOM trade statistics, Chapter HS.01, which covers all live animals, except for Fish and crustaceans, molluscs and other aquatic invertebrates which are included in Chapter HS.03, (i.e., 03.01, 03.06, 03.07, or 03.08) showed the approximate import values in United States dollars (USD) of the trade in these animals (Table 2). Of the 7 Caribbean countries Barbados had the highest import values, for pet animal ($371,604) and aquaria imports, $450,860 (Table 2).

**Table 2:** Trade analysis relevant to the Import Value of Live Animals and Aquaria Species in the Category [2006–2020].

Country	Total HS.01: Animals	Total HS.03: Fish & Crustaceans	Indicative Pet/Aquaria Imports in HS 01 and HS 03	
Antigua & Barbuda	$1,045,000		‘Pet’ imports dominated by turtles (HS: 01062010, 79 %), then live dogs (HS:01061910, 8.8 %) and other mammals (HS01061990)	$299,462
		$36,514,000	‘Aquaria’ dominated by cuttle fish and squid for breeding (HS:03074210, 55 %; then ornamental freshwater fish (HS:03011190 (1.7 %)	$157,418
Dominica	$1,019,000		‘Pet’ imports dominated by dogs (HS: 0106901, 88 %), then cats (HS: 01061020); turtles (HS:01062010) each averaging 5.6 % and 4.7 % respectively	$8180
		$7,924,000	‘Aquaria pet’ imports dominated by ornamental freshwater fish other than for breeding (HS:03011190, 63 %) and for breeding (HS:01011110, 36 %)	$12,199
Grenada	$4,859,000		‘Pet’ imports dominated by dogs (HS: 0106901, 85 %), then cats (HS:01061020, 9 %)	$316,101
		$17,068,000	No data could be differentiated for ‘Aquaria’ in the total HS 02 category	
St. Kitts & Nevis	$393,000		‘Pet’ imports dominated by undefined live mammals (HS:01061990, 69 %), then live Psittaciformes (parrots, parakeets, macaws, HS: 01063200, 10 %), turtles (HS:01062010, 9 %) and insects (excluding bees, HS: 01064900, 7 %)	$7397
		$19,657,000	‘Aquaria pet’ imports dominated by ornamental freshwater fish other than for breeding (HS03011190, 91 %), ornamental other than freshwater fish, not for breeding (HS:0301199o, 7.6 %)	$30,881
St. Lucia	$1,071,000		Pet’ imports dominated by dogs (HS: 0106901, 92 %), followed by cats (HS: 01061020, 5 %)	$20,549
		$138,663,000	‘Aquaria pet’ imports dominated by ornamental freshwater fish other than for breeding (HS: 03011190, 97 %)	$37,518
St. Vincent & the Grenadines	$588,000		‘Pet’ imports dominated by dogs (HS0106901, 91 %)	$7047
		$9,718,000	No data could be differentiated for ‘Aquaria’ as part of the total HS 03	
Barbados	$3,065,000		‘Pet’ imports dominated by dogs (HS0106901, 79 %), followed by live Psittaciformes (incl parrots, parakeets, macaws, HS: 01063200, 8.3 %)	$371,604
		$99,493,000	‘Aquaria’ imports considered pets, dominated by live ornamental fish for breeding (HS03011110, 91 %)	$450,860

### 3.2 In-country data gathering

#### 3.2.1 Size of pet aquaria trade industry

The largest number of businesses were found in Barbados with a total of 29 businesses (Table 3).

**Table 3:** Size indication of pet/aquaria trade in the OECS & Barbados based on stakeholder interviews.

Country	Business Type (private sector only)	No. of Businesses/ Operators Identified
Antigua & Barbuda	Pet Shop Pet Care service Animal Rescue & Sanctuaries Wildlife/nature parks	3 2 4 1
Dominica	Pet Shop	1
Grenada	Pet Shops Pet Care service Animal Rescue & Sanctuaries	5 3 1
St. Kitts & Nevis	Pet Shops (incl. importers and breeders)	5
St. Lucia	Pet Shop (breeders)	2
St. Vincent & the Grenadines	Pet Shop Pet Care service	1 4
Barbados	Pet shops and Pet Care Sanctuaries and Zoos	23 6

Source: Collated from the stakeholder identification process undertaken through in-country data gathering in 2022. ^a^Note this is not an exhaustive list; there may be several other operators that do not import, but purchase animals from their larger counterparts. A more comprehensive identification and mapping will need to be undertaken in collaboration with the Veterinary Division and Customs, to confirm the nature, distribution and level of market share and integration of the operators and to enable ‘registering’ of these entities for more effective private sector IAS management capacity building and risk communication associated with the pet and aquaria trade. Customs is particularly important to this exercise as based on the information provided by pet shop operators, Trinidad and Tobago appears to be a major source of exotic animals.

#### 3.2.2 Private sector feedback

The private sector feedback in select OECS countries revealed a diverse range of species being imported for pet and aquaria trade including fish, birds [(lovebirds), finches, parrots, macaws, songbirds)], and small mammals (Table 4). Key trends included angelfish and clownfish, live coral fragments, and finches were common in Dominica and Grenada (Table 4). Detailed pet import procedures and frequencies were noted, with an opportunity for improvement in biosecurity (no live fish inspection), a demand for species that are either not imported or are disallowed due to regulatory or environmental concerns (e.g. catfish, piranhas and capuchins, Table 4). Awareness levels varied, but there is recognition of potential IAS within countries (Table 4).

**Table 4:** Pet shop operator responses on imported pet/aquaria obtained from selected Organisation of the Eastern Caribbean States (OECS) countries.

Country	No. of Listed Firms	No. of Listed Firms contacted	No. of Listed Firms responding to survey	Species Imported	Import Process	Customer demand for species not imported or dis-allowed?	Aware of presence of prohibited/potentially invasive animals in country
Antigua & Barbuda	9	1	1	Fish: angelfish, clownfish; corals (life frags), goldfish, guppies (the latter two are more popular due to their ease of access/availability and decrease in prices)	Frequency: Fish imported monthly in peak season (summer); quarterly otherwise.	Catfish and piranhas Refuse to import species that may pose environmental danger	
Barbados	23	2	0	N/A	N/A	N/A	
Dominica	1	1	1	Dogs Birds: finches, lovebirds, cockatiels Fish: angelfish, barbs, bettas, bottom feeders, cherry shrimp, cichlids (mixed), crayfish, electric blue acaras, fancy guppies, goldfish, hilleries, koi, mollies, tetras platies, rams red-eared sliders (turtles)	Frequency: Fish - approx. every 2 months; Small birds every 4 months; Hamster once/year Pathway: Air and sea cargo (fish) sea cargo (accompanied) for hamsters & guinea pigs Procedure: Import permit required for all. Protocol includes quarantine inspection for birds, hamster & turtles. Live fish imports are not inspected. Did own RA for red-eared sliders	For more fish varieties, e.g., increased interest in Japanese koi karps for ornamental fish-ponds and tilapia for food Monkeys (capuchins), not allowed Tortoise and cats - bred locally	Cuban tree frog and Puerto Rican lizard
Grenada	2	2	1	Fish: goldfish, tetras, guppies, platies, hilleries, angelfish, gouramies, bettas, koi, mollies, catfish, danios, plecos, corydoras, other Birds: finches, lovebirds, cockatiels Guinea pigs Terrapins (red-eared sliders) Very few local breeders so import is only source for consistent supplies.			Amazon parrots (birds), tilapia and lionfish (fish), and brown anole (reptile), and the red millipede
St. Lucia	2	2	2	N/A	N/A	N/A	
St. Kitts & Nevis	4		1	Fish: goldfish, koi carp, molly, tetra barb, shark, Siamese fighter, zebra danio Cumberland slider turtle Guppy and tilapia bred locally	Frequency: fish - approx. every 4 months (pre-COVID) Pathway: Air cargo and private aircraft. Some air carriers do not allow live species transport Procedure: Import permit. None of the fish imported require an RA;	Piranhas which are prohibited, but does import pacu, similar in appearance.	Lionfish and snails (unsure of the tye of snail)
			2	Rabbit, guinea pigs Birds: geese; guinea fowl, pea fowl (peacock), quail, sensi fowl	Frequency: Approx. 1×/year Pathway: Sea cargo and through courier companies Procedure: Import permit required; Standard protocols apply; some animals may need quarantine once they enter. Own online research on their disease resistance and capacity to acclimatize locally	Toucan and claw duck (hybrid) from Guyana denied entry as well as some bird species from St. Martin	
			3	Import for self-use and occasional sale	Fish: goldfish, koi, guppy, molly Frequency: approx. 3×/year Pathway: Air and sea cargo Procedure: Import permit required. Does own online research on species before importing		
St. Vincent and the Grenadines	4	4	0	N/A	N/A	N/A	

N·B Not all operators that were contacted responded, and those that did response all did not agree to complete the survey. Those that did not agree to complete the survey did not think invasive alien species were relevant to their pet trading opweraitons.

#### 3.2.3 Importation source by country

Within the study sample for the OECS, Trinidad and Tobago was the most frequently identified Caribbean territory serving as a source country for pet and aquartia trade (Table 4). Barbados was next, followed by Guadeloupe and USA. Trinidad was the most frequently identified source country for aquarium fish then the USA. Birds were predominately sourced in Barbados and Trinidad and Tobago (Table 5).

**Table 5:** Main Pet/Aquaria imports and the relevant selected source country.

Country	Pet/Aquaria Imports	Source Country
Antigua and Barbuda	tropical aquarium fish	USA and Trinidad & Tobago
Dominica	dogs guinea pigs birds aquarium fish red-eared slider turtles	Barbados Guadeloupe and Trinidad & Tobago Barbados and Trinidad & Tobago Trinidad & Tobago Guadeloupe
Grenada	birds guinea pigs aquarium fish red-eared slider turtles	Barbados and Trinidad & Tobago Trinidad & Tobago Trinidad & Tobago Trinidad & Tobago
St. Kitts	birds aquarium fish	Barbados and Florida, USA Trinidad & Tobago

N·B Trade data by origin identifies all country sources. So if there was an intra-regional source, the island was named. Trinidad is the main Regional source for pets and all countries have a list of those which are allowed and excluded from trade. This does not cover illegal trade.

#### 3.2.4 IAS awareness within the industry (private sector)

While the responses from this limited survey under this study do not represent a sufficiently large sample size, the indications emerging appear to be consistent with the generally conclusion, as put by one OECS stakeholder, “*I think in general the public is clueless about IAS.*” A more focused country-based activity needs to build on this initial exercise in order to fill the critical information gaps to provide the evidence-base for awareness-building, risk communication and competency training.

The level of IAS management capacity and the level of sensitization among industry operators (private sector) about the potential IAS risks presented by their trade in, distribution of and ownership of animals for pets was generally high (Appendix A). This management capacity was for species not yet established or naturalized. The exception was a trader in St. Lucia who scored the lowest average score of 5 (Appendix A). Sensitization and risk communication across the pet and aquaria trade sector would be useful.

### 3.3 IAS risk prioritization tool design for the Organisation of the Eastern Caribbrean states (OECS) & Barbados

A process to develop a priority species list of IAS from pet and aquaria trade for the OECS and Barbados required a modified approach based on available tools, methodologies and existing knowledge from literature sources. Table 6 summarises the final criteria and associated questions used in the ranking process of invasive alien species (IAS) potential using a modified OHZDP or a One Health Invasive Alien Species Prioritization (OHIASP) tool during the *Regional Workshop on Preventing the COSTS of Invasive Alien Species in Barbados and the OECS: Successes, lessons learnt, and strategic partnerships in conserving biodiversity* from 20th-22nd March 2024. The focus of the OHIASP tool was IAS rather than zoonoses and the criteria were modified to reflect this.

**Table 6:** Final criteria and associated questions used in the ranking process of invasive alien species (IAS) potential using a One Health Invasive Alien Species Prioritization (OHIASP) during the workshop for Barbados & the OECS, 2024.

Ranking	Prioritization Criteria	Question	Answer (score)
1	Known invasive alien species	Is there evidence this animal is on a list of invasive alien species?	1 to 5 (lowest to highest)
2	Known in pet and aquaria trade	Is this animal known in the global pet/aquaria trade?	1 to 5 (lowest to highest)
3	Trending or Popularity	What is the popularity of this animal or is it trending?	1 to 5 (lowest to highest)
4	Public health impact	How likely is this animal to host zoonotic pathogens to cause human infections?	1 to 5 (lowest to highest)
5	Agricultural impact	How likely is this animal to cause agricultural damage?	1 to 5 (lowest to highest)
6	Ease of concealment or smuggling potential	How easy is it to conceal or smuggling this animal across borders?	1 to 5 (lowest to highest)
7	Establishment potential	How adaptable is this animal to existing ecosystems given its fecundity, lack of natural predators, etc.?	1 to 5 (lowest to highest)
8	Environmental impact	How likely is this animal to cause ecosystem and environmental damage?	1 to 5 (lowest to highest)
9	Economic impact	What is the potential negative economic impact of this animal if released?	1 to 5 (lowest to highest)
10	Eradication methods available	How available are methods to eradicate this animal?	1 to 5 (lowest to highest)
11	Distribution/Occurrence	What is the distribution or occurrence of this animal?	1 to 5 (lowest to highest)
12	Local/Global availability	How available is this animal to acquire from global sources (online)?	1 to 5 (lowest to highest)

N·B For each IAS, responses to each question are inputted into the adapted OHZDP tool for IAS risk assessment. Based on available evidence of IAS in the Caribbean or similar ecosystems and climate conditions, the relevant risk factor was assigned (High level of risk – 5 to low-no level of risk – 1) according to published literature reports, Google Scholar searches and or expert opinion of workshop participants. Each IAS was scored for each criterion based on collective national expertise and experience, Google scholar searches, published literature reports to evaluate risk with a minimum of 1 and a maximum of 5. These criteria were modified from OHZDP tool to reflect the substitution of zoonoses with IAS as the focus of the prioritization exercise and were developed and agreed upon during the regional IAS workshop in St. Kitts and Nevis.The key IAS prioritization steps are suggested below and presented as a matrix (Fig. 2). This matrix would permit development of ratings for each species, and shortlisting of species with the highest ratings. However, validation of the final IAS list was subject to local and regional expert validation.

### 3.4 Final scoring and ranking of IAS from pet and aquaria trade – Barbados & the OECS

Table 7 presents the final scores and ranking of 13 priority IAS from the pet and aquaria trade, with several ornamental freshwater fish and shellfish species as grouped as ‘freshwater ornamental fish’ and ‘freshwater ornamental shellfish’ respectively. The priority IAS list was finalised allowing presentation to the regional and national agencies. Planning for action, capacity building and monitoring will follow, supported a multisectoral One Health approach for Barbados and the OECS.

**Table 7:** Prioritization of IAS from the pet and aquaria trade in Barbados and the OECS with relevant prioritization criteria, total risk scores and ranking of each animal.

Prioritization Criteria	Fish				Amphibian	Reptiles			Birds				Primates
	Freshwater Ornamental Shellfish	Freshwater Ornamental Fish	Piranhas	Catfish	Cuban tree frog	Red-eared sliders	Puerto Rican lizard	Brown Anole	Lovebirds	Amazon parrots	Macaws	Songbirds	Capuchins
Known invasive alien species	5	4	5	5	5	5	5	5	5	5	5	5	5
Known in pet and aquaria trade	5	5	4	5	5	4	4	5	5	5	5	5	4
Distribution/Occurrence	5	5	3	4	3	3	3	4	5	4	4	5	3
Local/global availability	2	5	4	4	3	5	5	5	5	5	5	5	5
Eradication method availability	2	4	3	4	3	3	3	5	4	4	4	4	2
Trending or Popularity	4	5	5	4	5	4	4	5	5	5	5	5	5
Establishment potential	3	4	5	4	5	4	3	5	5	5	5	5	4
Agricultural impact	3	1	2	4	3	2	2	2	4	5	5	4	5
Public health impact	5	5	5	5	5	4	5	5	5	5	5	5	5
Environmental impact	3	1	2	4	3	2	2	2	4	5	5	4	5
Economic impact	1	2	5	1	2	1	1	1	1	3	3	2	1
Ease of concealment/smuggling potential	4	4	4	2	5	3	4	5	5	5	5	5	2
**Total Risk Score**	**42**	**41**	**47**	**46**	**47**	**40**	**23**	**39**	**52**	**56**	**56**	**54**	**52**
**RANKING**	**9**	**10**	**6**	**8**	**6**	**11**	**13**	**12**	**4**	**1**	**1**	**3**	**4**

N·B The list of priority IAS as agreed by the multisectoral group of national and international experts during the regional IAS workshop were red-ear sliders, lovebirds, capuchins, ‘freshwater ornamental fish’, ‘freshwater ornamental shellfish’, piranhas, catfish, Cuban tree frog, Puerto Rican lizard, brown anole, Amazon parrots, macaws, and songbirds. The eradication method availability rating is as follows: 1- excellent eradiation methods available to 5 – no effective eradiation method available, and this was ascertained using peer reviewed literature, Google Scholar searches and expert opinion among workshop participants. The preliminary list of IAS species considered during this exercise is presented in Appendix 2.

## 4 Discussion

This study is a landscape analysis of management of current IAS in six [6] Caribbean countries in the OECS in addition to Barbados, from the pet and aquaria trade. It aimed to generate country- and region-specific data on IAS risk management for pet and aquaria trade within Barbados and the OECS, a region where data are scarce, using stakeholder questionnaires and an IAS workshop. It represents the first ever approach to IAS risk management of animals using a modified OHZDP, although another study utilised this approach for invasive plant species [38]. The modified OHZDP or OHIASP method permits relevant IAS risk quantification, ranking and IAS list creation which can be communicated through both the public and private sectors. This approach can enhance data collection, risk management, and data management processes to manage IAS hazards from the pet and aquaria trade, given the challenge of data paucity. The process drives exploration of different types of data, highlighting the need for relevant data collection and data management for future IAS risk management to inform national policy and procedures.

### 4.1 Pet and aquaria trade in Barbados and the OECS

There is a significant market demand for non-native species bred locally, which could drive illegal trade or increase local breeding efforts in the Eastern Caribbean. In general, there are strict import processes and permits required for many species, indicating robust regulatory frameworks. The variety of pet species being imported in the OECS and Barbados reflects the different preferences of the consumers and includes fish, birds, and small animals as observed before [39,40]. There is a need, with resource limitations for effective systems to monitor, evaluate and assess risks associated with the pet and aquaria trade within the Caribbean to avoid deleterious impacts from IAS entry and establishment.

### 4.2 Trade risks and demand

The pet and aquaria trade industry faces challenges with compliance with environmental regulations, meeting consumer demands and preventing the introduction of invasive species in Barbados and the OECS [41,42]. Effective use of trade data is crucial in identifying IAS imported, trade volumes, species traits, ecological compatibility and developing management strategies for pet and aquaria trade [43]. Social drivers, internet, social media, e-commerce, and digital currencies are crucial for data sharing, risk analysis, and communication to inform IAS policy and management [3,5,44]. Integrating digital, social, and human behaviours relevant to the pet/aquaria trade in the Caribbean into risk analysis, IAS policy and management is critical in this modern age. Use of biosecurity concepts will also aid in characterizing the appropriate dynamic threats and risks influencing IAS risk management [6,45]. This will necessitate capacity building and increasing risk intelligence in the OECS and Barbados and is achievable by employing a blend of specialized training, legal analysis, education, and international collaboration.

### 4.3 Regional IAS workshop – St. Kitts & Nevis

During the regional IAS workshop, a consensus of thirteen [13] priority IAS — Amazon parrots, macaws, songbirds, lovebirds, capuchins, piranhas, catfish, Cuban tree frog, ‘freshwater ornamental fish’, ‘freshwater ornamental shellfish’, red-ear sliders, brown anole, and Puerto Rican lizard, (listed in the order of highest to lowest ranking) — from pet and aquaria trade was developed for Barbados and the OECS. Rankings of these IAS followed the relative criteria allowing IAS prioritising for pet and aquaria trade. Parrots and macaws are of concern due to their potential to be protected under the Convention on International Trade in Endangered Species of Wild Fauna and Flora (CITES). They both carry the highest risk totals and IAS ranking (joint #1) especially given the global threat of avian influenza or ‘bird flu’ [46]. This illustrates that the OHIASP tool can be used in conjunction with national or regional OHZDP efforts.

### 4.4 Regional vulnerabilities

The Caribbean's vulnerability to hydrometeorological events including hurricanes, tropical storms and weather systems can damage existing infrastructure increasing the risk of pet escapes [17]. Trinidad and Tobago, is the most popular source for pet and aquaria species among the eastern Caribbean countries. Additionally, there is an illegal wildlife trade that extends into surrounding South American countries such as Venezuela and Suriname [47]. The geopolitical instability of Venezuela and its close geographical proximity to Trinidad and Tobago have led to migration flows, public health challenges, and wildlife and animal trade issues [48,49]. IAS management is made even more difficult with added zoonotic threats to the economy, agriculture and public health. Additionally, the territorial dispute between Guyana and Venezuela, has significant implications beyond geopolitical tensions, exacerbated by wildlife trafficking risks, IAS risks and zoonoses [50].

### 4.5 Potential solutions and recommendations

To mitigate the IAS threats posed by the pet and aquaria trade, various measures can be implemented including increased veterinary border inspections, stricter animal care regulations, and prohibition of keeping certain species [51]. The cyber environment plays a significant role in pet and aquaria trade and modern IAS management integrate it into its strategy [27]. Web trawling/harvesting tools can be used to monitor legal and illegal wildife trade data and citizens' science approaches can enhance IAS management [5,52,53]. Multi-purpose digital/mobile apps can be strategic both in IAS and comprehensive disaster risk management (CDM) empowering communities to participate [54]. This should include protocols for securing pets within secure enclosures [55], strengthen biosecurity systems [45], implement real-time monitoring systems [56], establish rapid response teams [57], and integrate measures into national disaster recovery plans. Future studies examining hydrometeorological events or disasters and IAS management are needed to develop suitable biosecurity strategies, policy frameworks and resilience. Our OHIASP tool can aid other Caribbean countries in prioritising IAS from the pet and aquaria trade to improve IAS management efforts. The OHIASP tool can be linked to OHZDP tools to prioritise zoonoses that IAS can contribute to an increased risk profile. For example, a pet Eurasian bird species that becomes an IAS then established within the Caribbean, may serve as potential amplifying hosts for West Nile virus (WNV), increasing the risk potential for WNV outbreaks.

### 4.6 Existing challenges and limitations

The pet and aquaria trade faces challenges in IAS risk management including coordination, communication, resource constraints, and balancing different interests such as generating commerce from the trading of wildlife as pets, the risks of entry of IAS species, costs of providing the human resources needed to effectively monitor ports of entry and cargo. This requires balancing interests, improving infrastructure and establishing food security, agriculture and public health links but inadequate data systems and untrained personnel hinder timely access to pet and aquaria trade. We acknowledge the study limitations including weak data and traceability, in-country operators' reluctance to participate in the survey and limited data processing time due to project deadlines. Despite these, it aims to identify and address key IAS threats in the pet and aquaria trade in Barbados and the OECS, enhancing the current IAS management infrastructure for regional integration.

## 5 Conclusions

This marks the first ever use of an OHIASP tool for quantifying and ranking IAS risks in pet and aquaria trade pathways. It highlights the importance of using multipronged One Health approaches in the Caribbean, incorporating indigenous expert knowledge, digital tools, and community ownership to achieve sustainable outcomes for IAS management in Barbados and the OECS.

## Funding

The IAS research and the IAS workshop in St. Kitts and Nevis were funded by 10.13039/100011150Global Environment Facility (GEF) and prepared for The Centre for Agriculture and Bioscience International (CABI) Project Activity: 1.1.2.2: Risk Assessment for Horticulture Trade developed and published under the project “Preventing the COSTS of IAS in Barbados and the OECS”.

## CRediT authorship contribution statement

**Kirk O. Douglas:** Conceptualization, Data curation, Formal analysis, Investigation, Methodology, Software, Supervision, Validation, Visualization, Writing – original draft, Writing – review & editing. **Diana Francis:** Conceptualization, Data curation, Methodology, Project administration, Supervision, Validation, Writing – original draft, Writing – review & editing. **Naitram Ramnanan:** Funding acquisition, Project administration, Resources, Supervision, Validation, Writing – review & editing.

## Declaration of competing interest

The authors declare the following financial interests/personal relationships which may be considered as potential competing interests:

Naitram Ramanan reports financial support was provided by CABI. Naitram Ramnanan reports a relationship with CABI that includes: employment. Corresponding author was contracted under a consultancy to conduct this research. If there are other authors, they declare that they have no known competing financial interests or personal relationships that could have appeared to influence the work reported in this paper.

## Figures and Tables

**Table:** 

Interview Query	Country				
	Antigua & Barbuda	Dominica	Grenada	St. Lucia A	St. Lucia B
How would you rate:					
a. Your own understanding about invasive alien species (IAS)	2	2	2	5	1
b. The level of general awareness about IAS across the industry?	4	4	5	5	3
c. Your own understanding of the main species of IAS threats in your country?	2	2	2	5	3
d. The level of understanding that the industry could be a direct or indirect pathway for entry of threats?	1	1	1	5	3
e. Your level of awareness of the current IAS policies and/or regulations governing the industry?	3	3	3	5	5
f. The threat that IAS pose to the biodiversity of your country?	1	2	1	5	1
**Average score**	**2.2**	**2.3**	**2.3**	**5**	**2.7**

[Scale: 1 = highest; 5 = lowest].

Source: compiled from the limited stakeholder questionnaire undertaken for the purposes of this study.

**Table:** 

Type of Animal	Class	Name
Terrestrial	Primates	African green monkey
		Red howler monkey
		Capuchins
	Birds	Eurasian collared dove
		Ring necked parakeet
		Finches
		Lovebirds
		Amazon parrots
		Macaws
		Toucan
	Reptiles	Jamaican iguana
		St. Lucian iguana
		Boa constrictors
		Ball python
		Red eared sliders
		Puerto Rican lizard
		Brown anole
	Amphibians	Cuban tree frog
Aquatic	Fish	Piranhas
		Catfish
		Lionfish
		Guppy
		Tilapia
		Hilleries
		Platies
		Gouramies
		Mollies
		Danios
		Tetra barbs
		Plecos
		Angelfish
		Koi carp
		Siamese fighter
	Crustaceans	Blue shrimp
		Cherry shrimp
